# Treatment with Soluble Activin Type IIB Receptor Ameliorates Ovariectomy-Induced Bone Loss and Fat Gain in Mice

**DOI:** 10.1007/s00223-021-00934-0

**Published:** 2022-01-13

**Authors:** Tero Puolakkainen, Petri Rummukainen, Vappu Pihala-Nieminen, Olli Ritvos, Eriika Savontaus, Riku Kiviranta

**Affiliations:** 1 Institute of Biomedicine, University of Turku, Kiinamyllynkatu 10, 20520 Turku, Finland; 2 Department of Endocrinology, Division of Medicine, University of Turku and Turku University Hospital, Turku, Finland; 3 Department of Physiology, University of Helsinki, Helsinki, Finland; 4 Clinical Pharmacology, Turku University Hospital, Turku, Finland; 5 Turku Center for Disease Modeling, Institute of Biomedicine, University of Turku, Turku, Finland

**Keywords:** Activin, Osteoporosis, Histomorphometry, Ovariectomy, Glucose

## Abstract

**Introduction:**

In postmenopausal osteoporosis, hormonal changes lead to increased bone turnover and metabolic alterations including increased fat mass and insulin resistance. Activin type IIB receptors bind several growth factors of the TGF-β superfamily and have been demonstrated to increase muscle and bone mass. We hypothesized that ActRIIB-Fc treatment could improve bone and muscle mass, inhibit fat accumulation, and restore metabolic alterations in an ovariectomy (OVX) model of postmenopausal osteoporosis.

**Materials and Methods:**

Female C57Bl/6 N mice were subjected to SHAM or OVX procedures and received intraperitoneal injections of either PBS or ActRIIB-Fc (5 mg/kg) once weekly for 7 weeks. Glucose and insulin tolerance tests (GTT and ITT, respectively) were performed at 7 and 8 weeks, respectively. Bone samples were analyzed with micro-computed tomography imaging, histomorphometry, and quantitative RT-PCR.

**Results:**

Bone mass decreased in OVX PBS mice compared to the SHAM PBS group but ActRIIB-Fc was able to prevent these changes as shown by µCT and histological analyses. This was due to decreased osteoclast numbers and function demonstrated by histomorphometric and qRT-PCR analyses. OVX induced adipocyte hypertrophy that was rescued by ActRIIB-Fc, which also decreased systemic adipose tissue accumulation. OVX itself did not affect glucose levels in GTT but ActRIIB-Fc treatment resulted in impaired glucose clearance in both SHAM and OVX groups. OVX induced mild insulin resistance in ITT but ActRIIB-Fc treatment did not affect this.

**Conclusion:**

Our results reinforce the potency of ActRIIB-Fc as a bone-enhancing agent but also bring new insight into the metabolic effects of ActRIIB-Fc in normal and OVX mice.

## Introduction

During aging, the continuous decline in ovarian function leads to decreased secretion of sex hormones, including 17β-estradiol (E2), ultimately leading to menopause. As estrogen receptors are expressed in all different bone cell types, disturbances in E2-dependent signaling have been shown to impair osteoblastic functions and simultaneously promote osteoclast activity [1, 2]. Moreover, when E2 signaling is decreased, follicle-stimulating hormone (FSH) levels increase, which may also affect bone cells [3]. These events lead to accelerated, imbalanced bone turnover that favors resorption over formation resulting in bone loss [4, 5]. Ultimately, postmenopausal osteoporosis is characterized by skeletal fragility that is associated with increased risk for fractures.

Menopause has also significant metabolic sequelae. The decrease in estrogen and progesterone levels results in increased whole-body and liver adiposity as well as impaired glucose metabolism. However, the exact mechanisms behind these effects are incompletely understood. Menopause is strongly associated with lower energy expenditure, which increases the accumulation of adipose tissue at specific sites, decreases fatty acid oxidation, and favors adipocyte lineage commitment over osteoblasts in their common progenitor mesenchymal stem cells in the bone marrow [6–8]. Furthermore, the metabolic changes in menopause also advance systemic inflammation as the accumulation of free fatty acids and increased adipocyte cell death trigger TL4-dependent inflammatory responses. The inflammatory responses are thought to promote the development of insulin resistance and subsequently type II diabetes [9–11]. These alterations also promote the development of liver steatosis that may further aggravate inflammation and enhance gluconeogenesis in the liver, worsening the hyperglycemia [12].

Activins are systemic growth factors belonging to the Transforming Growth Factor β (TGF-β) superfamily and are best known for regulating the secretion of follicle-stimulating hormone (FSH), an essential regulator of growth and development [13]. Recent work has highlighted the role of activins and activin receptors in bone biology [14–16]. Activin A, the most abundant activin in bone, has been shown to context-dependently enhance receptor activator of NFkB ligand (RANKL)-induced osteoclastogenesis [17, 18]. Activin A also suppresses osteoblast differentiation and bone formation [19, 20]. Activin molecules bind to activin type II receptors, which activate the downstream signaling cascade. The inhibition of activins and other activin type II receptor ligands using soluble activin type II receptor fusion proteins, ActRIIA-Fc and ActRIIB-Fc, respectively, have been demonstrated to increase bone mass and quality in multiple animal models [16, 21–23]. In addition, ActRIIB-Fc has been shown to decrease adipocyte size and reduce accumulation of white adipose tissue (WAT) [24]. Koncarevik et al. reported that ActRIIB-Fc treatment protects against the systemic effects induced by androgen deprivation, including gain of adipose tissue and liver steatosis [25]. Collectively, these reports demonstrate ActRIIB-Fc ligands to be important regulators of muscle, bone, and adipose homeostasis. As the global prevalence of osteoporosis, aging-related frailty as well as the menopause-associated metabolic syndrome is rising, we set out to investigate whether the treatment with ActRIIB-Fc could modulate both bone and metabolic effects of estrogen deprivation in an ovariectomy mouse model, a widely accepted animal model. We hypothesized that treatment of ovariectomized (OVX) mice with ActRIIB-Fc would protect against the negative effects of estrogen deprivation by maintaining or even increasing bone mass and quality, inhibiting the site-specific white adipose tissue (WAT) accumulation and by maintaining/improving glucose metabolism.

## Materials and Methods

### Production of ActRIIB-Fc

The production of this fusion protein has been reported previously [26]. In brief, the ectodomain of human ActRIIB and the human IgG1-Fc domain were amplified and subcloned into an expression vector. The expression vector was transfected into Chinese hamster ovary cells, which were grown in a suspension culture.

### Animals

7- to 9-week old female wild-type C57Bl/6 N mice (Envigo, Netherlands) were used in this experiment. The mice were kept in cages in groups of four to five under standard laboratory conditions (temperature 22 °C, light cycle 6:00AM–6:00PM). The animals were given water and soya-free food pellets (RM3-E, Special diets services, UK) ad libitum excluding fasting periods before glucose and insulin tolerance tests and euthanization. The food pellets were stocked and weighed every week to indirectly measure caloric intake of the mice. In addition, spillage was accounted for by checking the cages for food crumbs.

### Study Design

The mice were divided into four groups and were subjected to either sham-surgery or ovariectomy (OVX). The animals were allowed to rest 1 week post-operatively, after which mice were given intraperitoneal (i.p.) injections of either phosphate-buffered saline (PBS) or ActRIIB-Fc (5 mg/kg) once a week for 7 weeks. The dose was based on our previous study where no differences in body or muscle mass were noted between the groups receiving either 5 mg/kg or 10 mg/kg injections [26]. Quantitative nuclear magnetic resonance (NMR) was used to assess body composition during weeks 1 (before treatment), 5, and 8. A glucose tolerance test was performed 8 days and an insulin tolerance test 1 day before euthanization. Calcein and demeclocycline i.p. injections were given 10 and 3 days, respectively, before the mice were killed by CO2 asphyxia and cervical dislocation. Four mice were omitted from the study as they developed rashes or post-operative complications. Group sizes were as follows: *n* = 10 for SHAM PBS group, *n* = 9 for SHAM ActRIIB-Fc group, *n* = 18 for OVX PBS group, and *n* = 18 for OVX ActRIIB-Fc group.

### Surgical Procedure

Under isoflurane anesthesia (250–400 ml/min 2.5%) and aseptic surgical conditions, subcutaneous injections of carprofen (5 mg/kg) and buprenorphine (0.05 mg/kg) were given. A midline incision was done in the mid-dorsum of the mouse. The cutaneous and subcutaneous tissues were then gently separated laterally from the underlying muscle tissue. Then a small incision was done over the muscle layer to expose the ovary. The ovary was then gently raised and removed from the surrounding fat tissue by cauterization. The ovarian horn was then released back into the peritoneum. A self-resorbing suture was placed over the muscle layer, and a non-resorbing suture was set on the skin. The procedure was repeated on the contralateral side. The sham procedure was done as above except after raising the ovaries from their surroundings, and they were gently put back into place and left intact. The mice were administered with post-operative injections of carprofen and monitored regularly for the next 2–3 days.

### Body Composition Analysis

Whole body composition was measured by quantitative NMR (EchoMRI-700, Echo Medical Systems) as previously reported [27]. The mice were restrained to a transparent cylinder tube to ensure immobility during the scan. The scan was performed twice and mean values for fat and lean mass were calculated and then adjusted according to the body weight of each individual animal.

### Body Weight and Tissue Sample Weights

During the experiment, the animals were weighed with a high-quality scale that was calibrated before each use. The animals were weighed before the surgical procedure, each injection, each NMR scan, and euthanization.

After the mice were sacrificed WAT depots (gonadal, retroperitoneal, mesenteric), the scapular BAT depots, livers, and the uteri were weighed immediately after dissection with a high accuracy analytical scale.

### Micro-computed Tomography Analyses

The detailed specifications of the micro-computed tomography analyses have been previously reported [23]. Briefly, X-ray microcomputer tomography, µCT (Skyscan 1070, Kontich Belgium) was used to image and analyze the bone structure of the cortical and trabecular bone of the distal femur and the 2nd lumbar vertebrae. After scanning, the images were reconstructed (Nrecon 1.4, Skyscan) with identical settings. The regions of interests (ROI) were drawn (CTan 1.4.4, SkyScan), and the results were then quantified and analyzed. The ROIs for each sample were drawn blinded. The data were then quantified and subjected to statistical evaluation according to current guidelines [28].

### Histological and Histomorphometrical Analyses

For histological analyses the femur, gonadal WAT, BAT and liver samples were fixed in formalin, (femurs were decalcified in 10% EDTA), embedded in paraffin and then cut into 5 µm sections using a high-quality microtome. The sections were then deparaffinized, rehydrated and stained with hematoxylin & eosin (femur, WAT, BAT, liver), or tartrate-resistant acid phosphatase (femur) staining, respectively, according to standard protocols. Cryosections of snap-frozen liver samples were cut with a cryostat and stained with Oil-Red-O (ORO) to measure liver adiposity. For histomorphometry the tibiae were fixed with 3.7% formalin in PBS, dehydrated in 70% EtOH and embedded in methyl methacrylate (Sigma-Aldrich). 54 µm sagittal sections were cut using a Leica RM2255 microtome and then Von Kossa, Toluidine blue and TRACP-staining were performed on the de-plastified sections with standard protocols.

The bone slides were analyzed using an Osteomeasure-histomorphometry work station (Osteometrics, USA). The analyzed area of the femur was defined as approximately 800 µm × 1000 µm starting 300 µm proximally from the distal growth plate excluding the cortical bone borders. The trabecular islands and the TRACP-stained areas were drawn and quantified. The fat droplets located within this region of interest were also quantified to assess bone marrow adiposity. Histomorphometrical analysis of the tibiae was done on a standard sampling site, 200 µm below the growth plate on an area of 1.17mm^2^. Quantitative measurements were performed semiautomatically according to standardized protocols [29].

In the gonadal WAT samples, adipocyte diameters were calculated by measuring the diameter of each adipocyte from two perpendicular angles (*n* = 30–50 adipocytes per sample) using ImageJ image processing program (version 1.51 g) and then calculating the mean of these values. The adipocyte size distribution was also computed.

Liver adiposity was analyzed blinded by choosing three representative areas of each ORO-stained liver sample. Quantification of adipocyte droplets was done using ImageJ by setting the threshold values so that only ORO-stained droplets were accounted for in the analysis. The numerical value for each corresponding sample was calculated by taking the mean number of droplets from the three areas.

### Glucose and Insulin Tolerance Tests

Glucose and insulin tolerance tests (GTT and ITT, respectively) were performed on the animals to evaluate changes in glucose metabolism as previously described [27]. For GTT, the animals were allowed to fast for four hours and were then administered an individual dose of 5% glucose solution (1 g/kg) i.p. The blood glucose levels were measured from the lateral tail vein immediately before injection and 20, 40, 60, and 90 min after the injection using Precision Xtra glucose monitoring device (Abbott Diabetes Care, Abbott Park, USA). For ITT, the subjects were allowed to fast for one hour and were then injected i.p. with human insulin 1.0 IU/kg. The blood glucose levels were measured right before injection and 20, 40, and 60 min after.

### Serum Sample Analyses

Blood samples were collected via exsanguination by cardiac puncture. Samples were then let stand followed by centrifugation to separate the serum, which was then stored at − 80 °C. Serum levels of C-terminal telopeptide (CTX) and N-terminal type I procollagen (P1NP) were analyzed by ValiRx laboratory, Finland with RatLaps (CTX-1) EIA and Rat/Mouse P1NP EIA assay kits, respectively.

### Measurement of Gene Expression

Detailed specifications of the quantitative real-time PCR results have been published earlier [23]. Total RNA from homogenized, marrow-free, bone samples was isolated using TriSure reagent followed by DNAase treatment and RNA clean-up using RNeasy minikit (Qiagen, Germany). The cDNA was then synthesized from 1 µg of RNA with SensiFAST probekit (Bioline, UK). Quantitative real-time PCR was performed using iQ SYBR Green Supermix (Bio-Rad laboratories, USA). *β-actin* was used as the internal control*.* Primer sequences are available upon request.

### Statistical Analyses

All of the quantified data were then subjected to repeated measures (RM)-ANOVA, to assess significance over time, or two-way ANOVA where “Treatment” refers to the effects of PBS or ActRIIB-Fc, “Surgery” refers to the SHAM or OVX surgical procedure, and “Interaction” refers to the interactive effect of both “treatment” and “surgery.” This was followed by the Tukey or Tukey–Kramer test as post hoc tests. Student’s *T* test was used when only two groups were compared. Results were considered statistically significant when *p* value was lower than 0.05. Statistical analyses were performed with IBM SPSS Statistics v.20 (IBM, USA).

## Results

### ActRIIB-Fc Treatment Increases Lean Mass and Decreases Systemic Adiposity

**Fig. 1:**
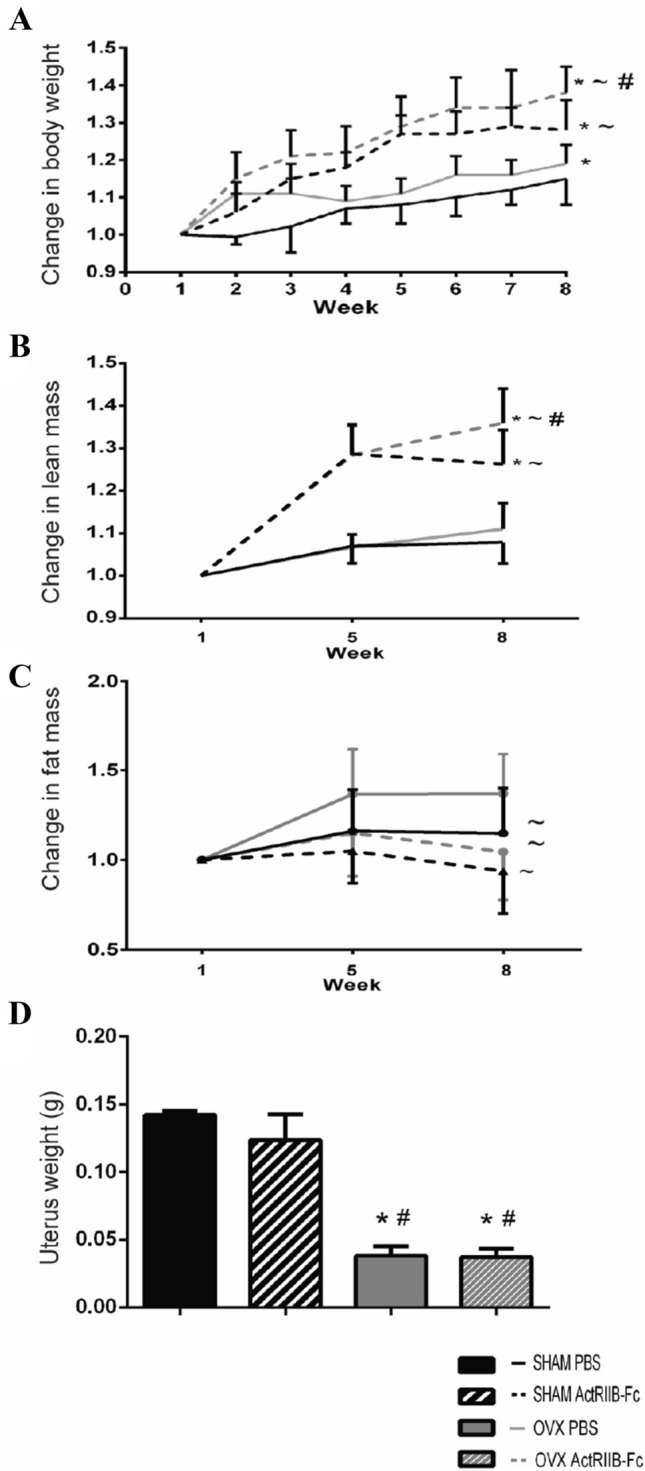
ActRIIB-Fc treatment increases lean mass and prevents fat accumulation. **A** Changes in body weight. ActRIIB-Fc robustly increased body mass during the experiment in both SHAM and OVX mice. **B** ActRIIB-Fc-treated mice had robust improvements in lean mass in both SHAM and OVX groups compared to their PBS-treated controls. **C** OVX increases systemic fat accumulation compared to PBS-treated SHAM controls. ActRIIB-Fc attenuated these changes in OVX mice and a similar trend was seen in SHAM mice as well. **D** OVX induced potent uterine atrophy compared to SHAM mice verifying the success of the surgical procedure. *p < 0.05 compared to SHAM PBS. ~*p* < 0.05 compared to OVX PBS. #*p* < 0.05 compared to SHAM ActRIIB-Fc. *n* = 9–10 for SHAM groups, 18 for OVX groups

As expected, ActRIIB-Fc treatment resulted in robust increases in body weight (Fig. 1A). Weights of the OVX ActRIIB-Fc group were 16% higher compared to OVX PBS group and 11% higher in SHAM ActRIIB-Fc group compared to SHAM PBS group, respectively (RM ANOVA *p* < 0.001 in both.)

The lean mass changes induced by OVX only slightly differed from the SHAM mice treated with PBS suggesting that the surgical procedure and the succeeding events did not have a great effect on the lean mass in mice (Fig. 1B). ActRIIB-Fc treatment robustly increased lean mass, as expected, by 22% in OVX mice and by 17% in SHAM mice compared to their corresponding PBS-treated groups (RM ANOVA *p* < 0.001 in both). This also suggests that most of the body weight that was gained during the experiment in ActRIIB-Fc-treated mice was in fact lean mass.

OVX resulted in increased systemic adiposity compared to PBS-treated SHAM controls (Fig. 1C). The effects of OVX on changes in fat mass were more evident during the first half of the experiment and then evened out during the second half of the study. Ultimately, the fat mass of the OVX PBS mice increased by 19% compared to SHAM PBS control group (RM ANOVA *p* = 0.006). ActRIIB-Fc treatment prevented this as treatment resulted in 24% less accumulated fat compared to the PBS-treated OVX mice (RM ANOVA *p* = 0.003). Similar changes, though not statistically significant, were noted in the SHAM mice as ActRIIB-Fc treatment resulted in 18% less fat accumulation compared to the SHAM PBS mice.

There were no differences in food intake between the groups of mice as measured by the weights of the restocked food pellets. Finally, as ovariectomy results in uterine atrophy, the uteri of each mouse were weighed to confirm the success of the OVX procedure (Fig. 1D). The uteri of the OVX mice were atrophied during the experiment compared to SHAM mice as expected (ANOVA Surgery *p* < 0.001).

### ActRIIB-Fc Attenuates Ovariectomy-Induced Changes in Bone Mass and Quality

**Fig. 2:**
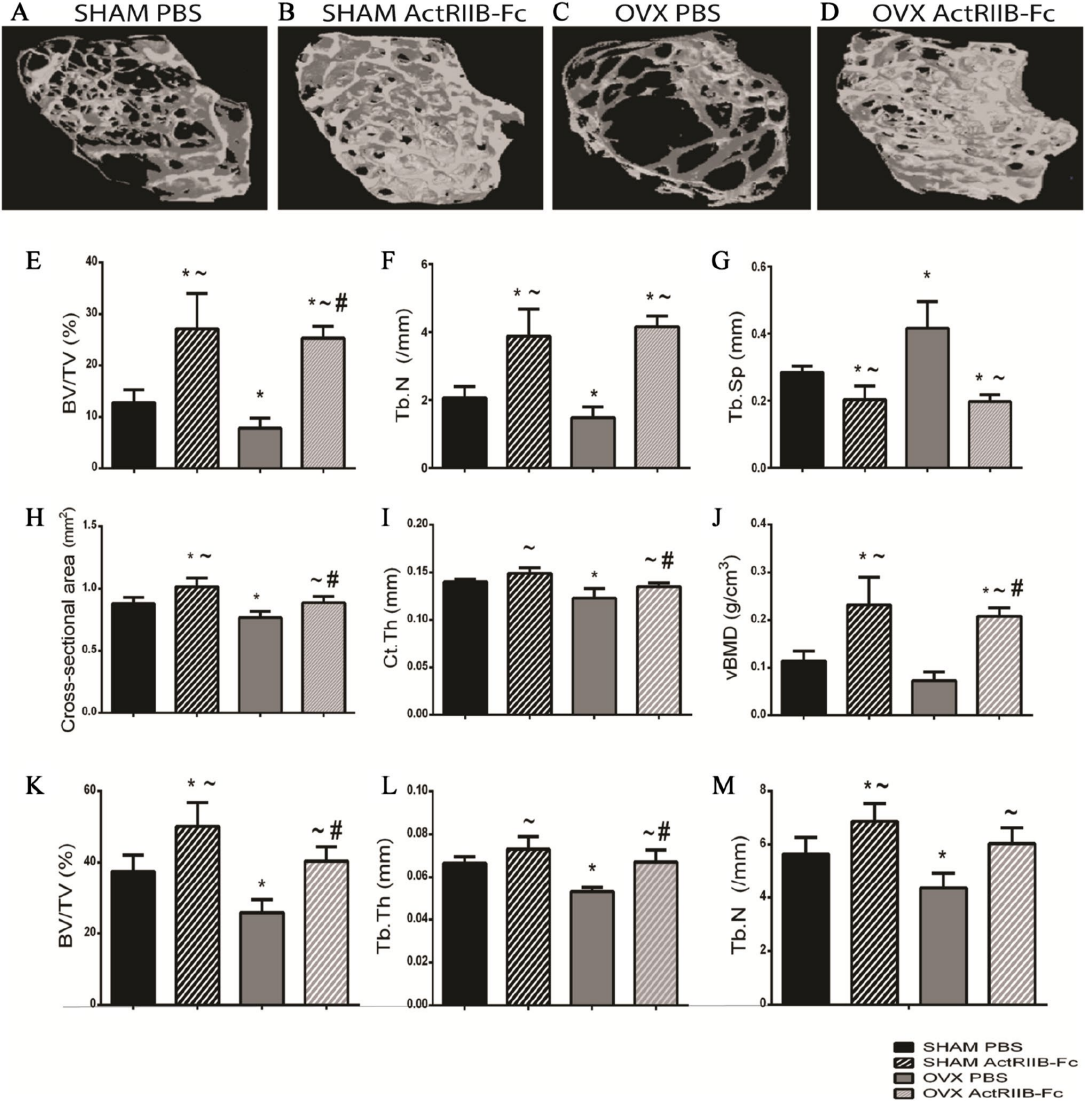
ActRIIB-Fc treatment ameliorates OVX-induced bone loss. Reconstructed 3D images of **A** SHAM PBS, **B** SHAM ActRIIB-Fc, **C** OVX PBS and **D** OVX ActRIIB-Fc trabecular bone in the distal femur. µCT analyses showed ActRIIB-Fc treatment increased **E** bone volume per tissue volume and **F** trabecular numbers while decreasing **G** trabecular separation in SHAM and OVX mice. Effects on cortical bone were more modest as ActRIIB-Fc mildly improved **H** mean cross-sectional bone area and **I** cortical thickness. ActrIIB-Fc-treated mice had also higher trabecular **J** volumetric bone mineral density. Similar results were seen in the 2nd lumbar vertebrae as ActRIIB-Fc treatment increased **K** BV/TV, **L** trabecular thickness as well as **M** Tb.N. *p < 0.05 compared to SHAM PBS. ~ *p* < 0.05 compared to OVX PBS. #*p* < 0.05 compared to SHAM ActRIIB-Fc. Femur: *n* = 9–10 for SHAM groups, 18 for OVX groups. Vertebrae: *n* = 8–9 for SHAM groups, 9–10 for OVX groups

µCT analyses were performed to assess the changes in bone mass and quality in the distal femur (Fig. 2A–D). As expected, OVX resulted in osteopenia as bone volume/tissue volume (BV/TV—39% ANOVA Surgery *p* < 0.001) (Fig. 2E) and trabecular numbers (Tb.N—28% ANOVA Surgery *p* < 0.001) (Fig. 2F) were significantly lower and trabecular separation (Tb.Sp + 46% ANOVA Surgery *p* < 0.001) (Fig. 2G) higher in OVX PBS mice compared to the SHAM PBS controls. ActRIIB-Fc treatment prevented the OVX-induced bone loss as BV/TV was improved by 220% and Tb.N by 180% and Tb.Sp decreased by 53% when comparing OVX ActRIIB-Fc group to OVX PBS mice. Similarly, ActRIIB-Fc had similar effects on the same parameters as above (+ 110%, + 88% and − 28%, respectively) in SHAM mice compared to the PBS-treated SHAM group (ANOVA Treatment *p* < 0.001 in all) (Fig. 2E–G). In addition, ActRIIB-Fc improved cortical parameters in OVX ActRIIB-Fc group as cross-sectional bone area was 16% higher and cortical thickness 10% higher compared to the OVX PBS group (ANOVA Treatment *p* < 0.001 in both) (Fig. 2H, I). ActRIIB-Fc also significantly increased cross-sectional bone area by 15% (*p* < 0.001) in SHAM mice but did not have a significant effect on cortical thickness. Furthermore, ActRIIB-Fc treatment robustly improved trabecular volumetric bone mineral density (vBMD) + 187% OVX ActRIIB-Fc vs OVX PBS group and + 102% SHAM ActRIIB-Fc vs SHAM PBS group ANOVA Treatment *p* = 0.006 (Fig. 2J.). ActRIIB-Fc did not significantly improve cortical bone vBMD in either group (data not shown.)

Similar results were observed in the 2nd lumbar vertebrae as the ovariectomized, PBS-treated mice had 31% lower BV/TV, 11% lower trabecular thickness (Tb.Th) and 23% lower Tb.N (ANOVA Surgery *p* = 0.001–0.004) compared to the SHAM PBS group (Fig. 2K–M). ActRIIB-Fc was able to prevent OVX-induced bone loss also in the vertebrae as BV/TV was 60% higher, Tb.Th 13% higher and Tb.N 40% (ANOVA Treatment *p* = 0.001–0.007) higher in OVX ActRIIB-Fc mice compared to the OVX PBS group. As expected, BVTV was 34% greater, Tb.Th 10% greater and Tb.N 20% greater in the SHAM ActRIIB-Fc group compared to the SHAM PBS controls.

### ActRIIB-Fc Decreases Bone Resorption by Affecting Osteoclast Numbers

**Fig. 3:**
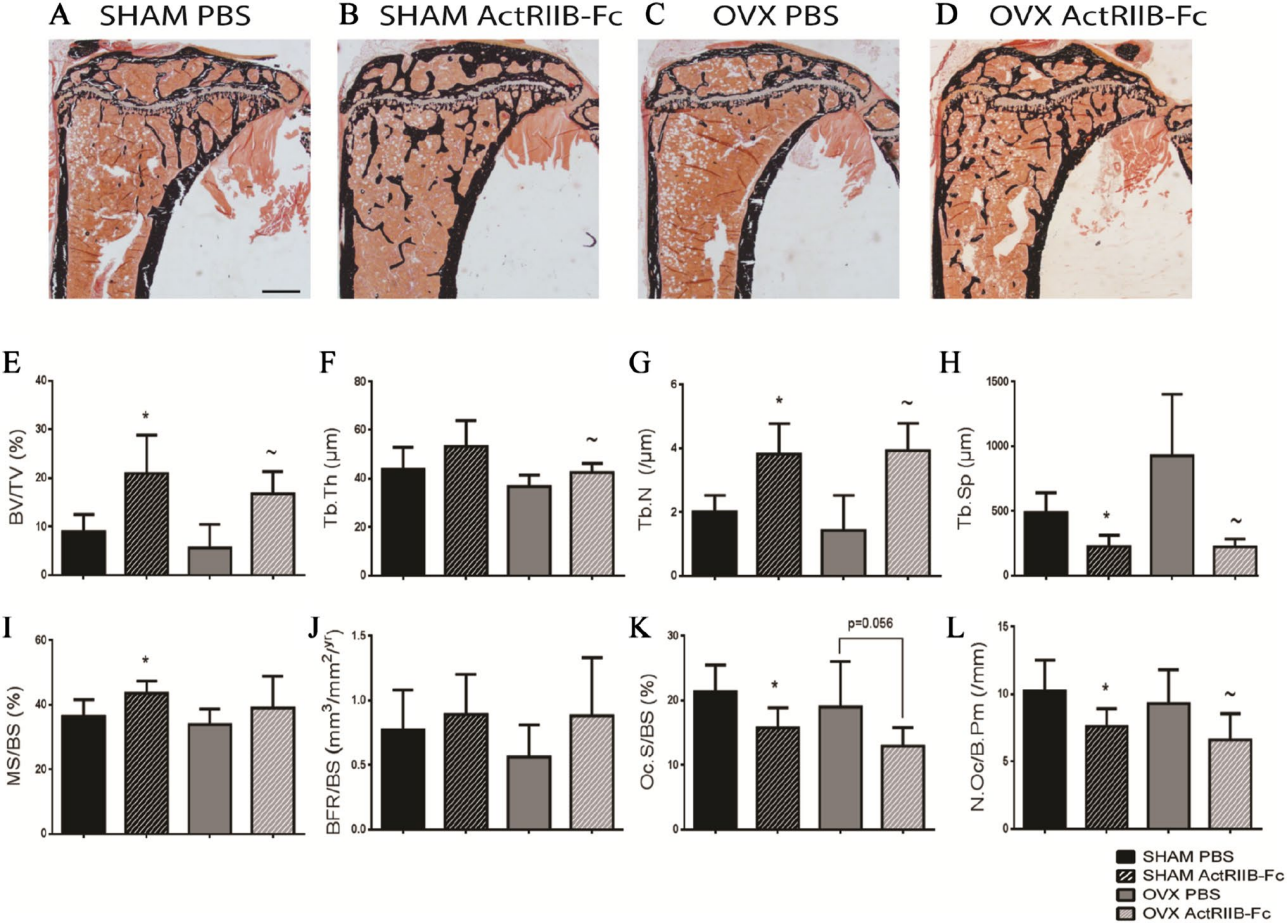
ActRIIB-Fc decreases bone resorption by affecting osteoclast numbers. Representative von Kossa-stained tibiae from **A** SHAM PBS, **B** SHAM ActRIIB-Fc, **C** OVX PBS, and **D** OVX ActRIIB-Fc groups. Histomorphometrical analyses verified structural changes induced by ActRIIB-Fc treatment by increasing **E** BV/TV, **F** Tb.Th, and **G** Tb.N and decreasing **H** Tb.Sp in both SHAM and OVX mice. Dynamic parameters suggest positive trends in (**I**) mineralized surface/bone surface as well as (**J**) bone formation rate per bone surface due to ActRIIB-Fc treatment. Treatment with ActRIIB-Fc also decreased (**K**) osteoclast surface per bone perimeter as well as (**L**) numbers of osteoclasts per bone perimeter. *p < 0.05 compared to SHAM PBS. ~ *p* < 0.05 compared to OVX PBS. #*p* < 0.05 compared to SHAM ActRIIB-Fc. *n* = 5–6 per group. Black bar = 400 µm

To further elucidate the cellular mechanisms behind these findings, we performed histomorphometric analyses on the tibiae (Fig. 3A–D). Structural analyses confirmed our µCT findings as ActRIIB-Fc treatment increased BV/TV by 130% and 200%, Tb.Th by 22% and 15%, Tb.N by 90% and 174% and Tb.Sp decreased by 53% and 76% when comparing to the corresponding SHAM and OVX PBS groups, respectively (ANOVA Treatment *p* < 0.001 in all, Fig. 3E–H). The changes seen in the dynamic parameters were not as evident. ActRIIB-Fc treatment increased the mineralizing surface/bone surface (MS/BS) in SHAM mice by 20% (ANOVA treatment *p* = 0.033) and by 15% in OVX mice (Fig. 3I). In addition, trends of increased bone formation rate per bone surface (BFR/BS) were noted in ActrRIIB-Fc groups compares to controls, but these were not statistically significant in (ANOVA Treatment *p* = 0.089) (Fig. 3J). As we had described previously in a fracture model [30], ActRIIB-Fc treatment decreased osteoclast surface per bone surface (Oc.S/BS) by 26% and 32% (ANOVA Treatment *p* = 0.029) as well as number of osteoclasts per bone perimeter (N.Oc/B.Pm) by 26% and 29% (ANOVA Treatment *p* = 0.007) when comparing to corresponding PBS-treated SHAM and OVX groups, respectively (Fig. 3K, L).

### ActRIIB-Fc Treatment Affected the Expression of Important Resorbing Genes Associated with Bone Formation and Resorption

**Fig. 4:**
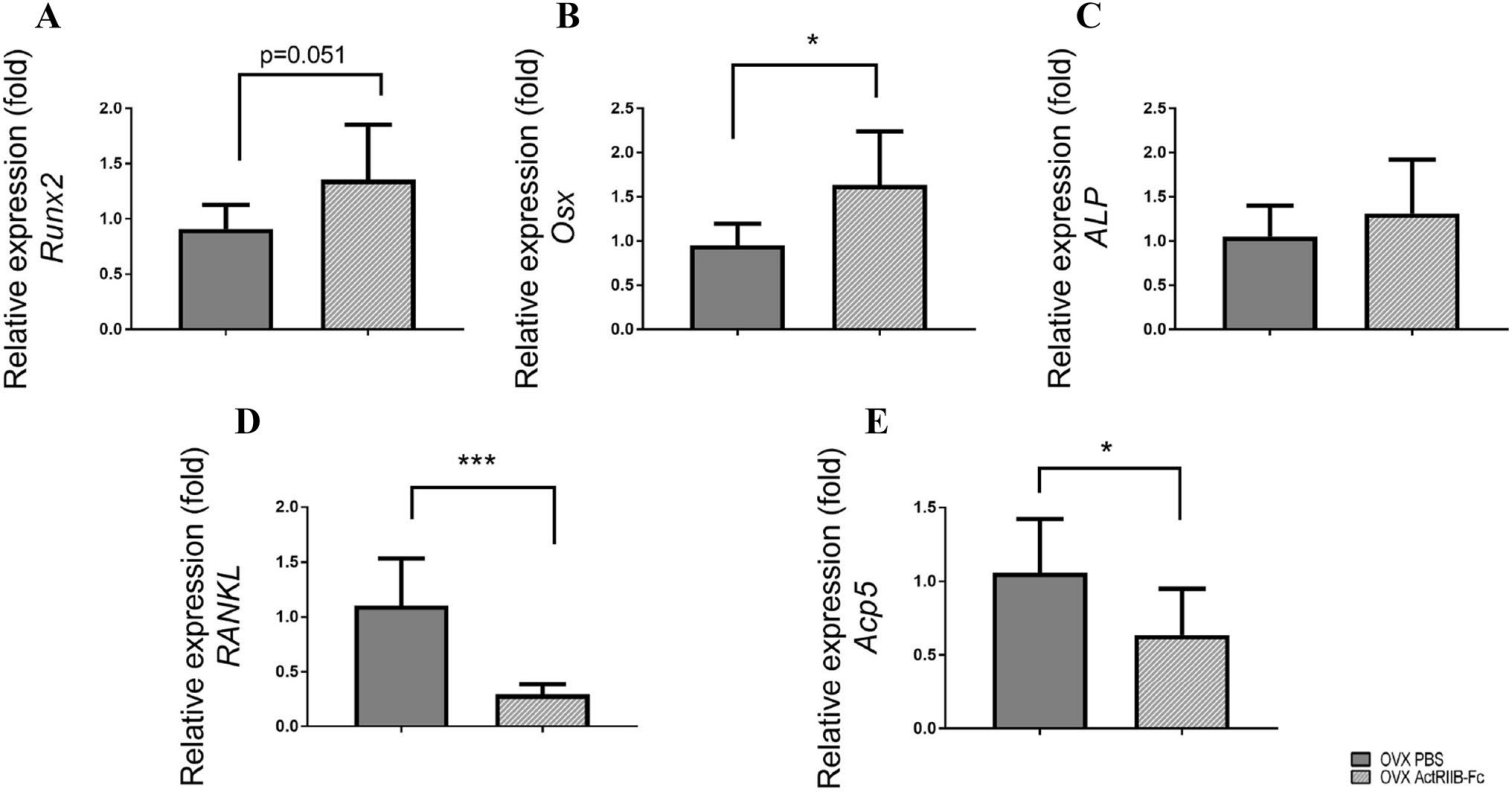
ActRIIB-Fc increases expression of osteogenic markers and decreases expression of resorption markers in OVX mice. Quantitative real-time PCR analyses show ActRIIB-Fc to alter expression of **A** Runt-related transcription factor-2, **B** Osterix and **C** Alkaline phosphatase 1 suggesting an anabolic response in bone. Decreased expression values of osteoclast markers **D** receptor activator of kappa-B ligand and **E** tartrate-resistant acid phosphatase in ActRIIB-Fc-treated OVX mice were also observed. *n* = 6–8 per group

To elucidate the mechanisms behind the effects of ActRIIB-Fc in OVX mice, we measured the expression of essential osteoblast and osteoclast genes in long bone samples using quantitative real-time PCR. ActRIIB-Fc treatment resulted in a modest increased expression of runt-related transcription factor-2 (*Runx2*) in the OVX mice (+ 49.0% *p* = 0.051) and Osterix (*Osx*) by 71.8% (*p* = 0.017) as shown in Fig. 4 A, B. No significant differences were observed in the expression of alkaline phosphatase (*Alp1*) (Fig. 4C). ActRIIB-Fc treatment significantly decreased the expression of activator of nuclear factor kappa-B ligand (*RANKL* − 73.9% *p* < 0.001) as well *Acp5*, a gene for tartrate-resistant acid phosphatase (− 39.9%, *p* = 0.019).

### ActRIIB-Fc Rescues OVX-Induced Adipocyte Hypertrophy

**Fig. 5:**
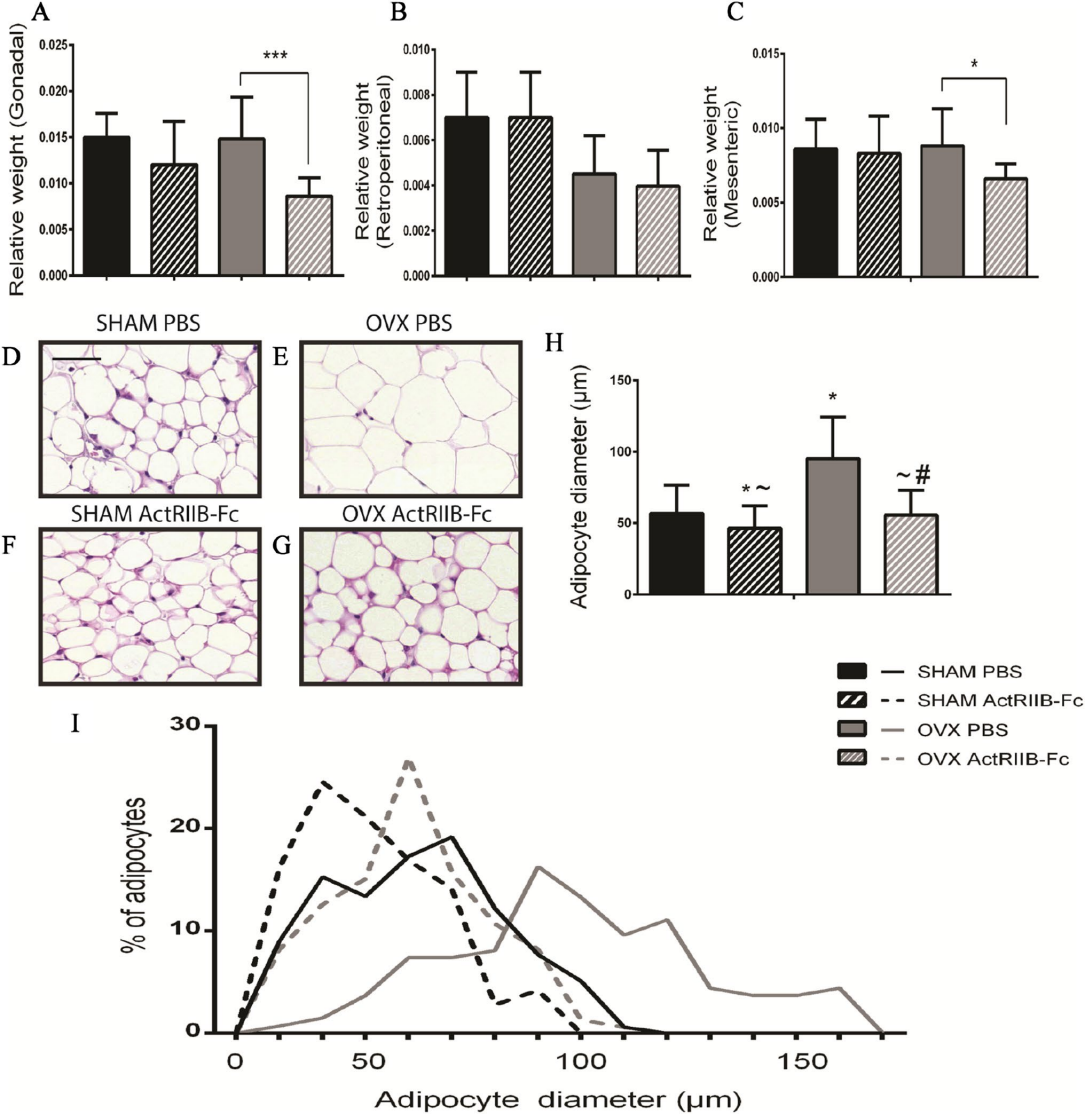
ActRIIB-Fc protects from OVX-induced fat gain. ActRIIB-Fc treatment decreases the relative weight of **A** gonadal fat pad in both OVX and SHAM mice. ActRIIB-Fc treatment did not affect the retroperitoneal fat pad (**B**). ActRIIB-Fc also decreased the relative weight of **C** mesenteric fat pad but only in OVX mice. Representative histological images of **D** SHAM PBS, **E** OVX PBS, **F** SHAM ActRIIB-Fc, and **G** OVX ActRIIB-Fc gonadal adipocytes. **H** ActRIIB-Fc treatment decreases adipocyte size in both SHAM and OVX mice compared to their corresponding groups. **I** Graph showing distribution of different adipocyte sizes. For fat pad analyses *n* = 9–10 for SHAM groups, 18 for OVX groups. For adipocyte diameters *n* = 120–160 per group. *p < 0.05 compared to SHAM PBS. ~*p* < 0.05 compared to OVX PBS. #*p* < 0.05 compared to SHAM ActRIIB-Fc. Black bar = 100 µm

As the NMR-body composition analysis indicated significant changes in the whole-body adiposity, we weighed WAT depots and adjusted the data to the body weights of each animal. Only small changes were seen when comparing WAT tissue depot weights between PBS and ActRIIB-Fc-treated SHAM mice. However, treatment with ActRIIB-Fc led to a 42% reduction in gonadal WAT weight (ANOVA treatment *p* = 0.007) and to a 25% reduction in mesenteric WAT weight (ANOVA treatment *p* = 0.051) compared to the PBS-treated OVX mice. The retroperitoneal fat depot appeared not to respond to ActRIIB-Fc treatment (Fig. 5A–C). In addition, body weight-adjusted scapular BAT depot weighs were slightly decreased due to ActRIIB-Fc treatment in both SHAM and OVX mice. However, there were no apparent histological changes in the morphology of BAT depots between the groups (data not shown).

To determine whether the decrease in the gonadal fat depot weights was due to reduction in adipocyte size we analyzed H&E stained sections to measure the mean diameter of the WAT adipocytes and the distribution of different size adipocytes within the tissue. As expected OVX induced adipocyte hypertrophy as the mean diameter of the adipocytes increased by 68% (Tukey’s test *p* < 0.001) in OVX PBS mice compared to the SHAM PBS controls. ActRIIB-Fc treatment blunted this effect as the mean adipocyte diameter was 42% (Tukey’s test *p* < 0.001) smaller in OVX ActRIIB-Fc mice compared to OVX PBS mice. Similar but less pronounced response was seen in the SHAM groups as treatment with ActRIIB-Fc resulted in 18% (Tukey’s test *p* < 0.001) smaller adipocytes compared to the SHAM PBS mice.

### ActRIIB-Fc Impairs Glucose Tolerance but Does Not Affect Development of Insulin Sensitivity

**Fig. 6:**
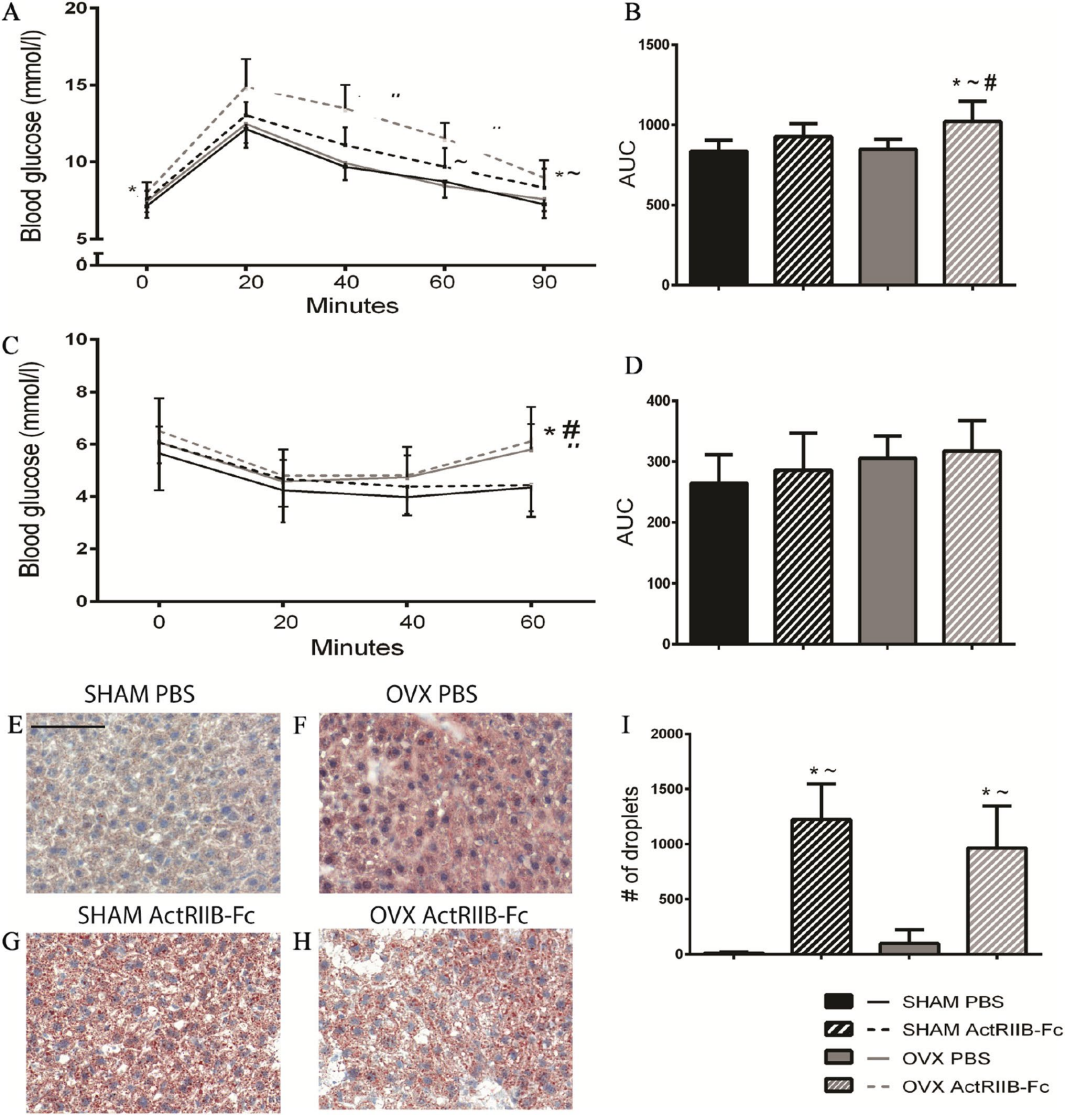
ActRIIB-Fc impairs glucose tolerance and induces hepatic steatosis. ActRIIB-Fc impairs glucose tolerance especially in OVX mice but similar trends are seen in SHAM mice as well as see in **A** Glucose tolerance test. The **B** area under the curve was also quantified. ActRIIB-Fc treatment had no effect on the development of insulin resistance as shown by **C** insulin tolerance test and the **D** area under the curve. ActRIIB-Fc induced hepatic steatosis as seen in representative oil-red-O stained liver sections of **E** SHAM PBS, **F** OVX PBS, **G** SHAM ActRIIB-Fc, and **H** OVX ActRIIB-Fc groups. **I** Quantification of adipose droplets verified these findings. *p < 0.05 compared to SHAM PBS. ~*p* < 0.05 compared to OVX PBS. #*p* < 0.05 compared to SHAM ActRIIB-Fc. GTT/ITT *n* = 9–10 for SHAM groups, 18 for OVX groups. Liver analysis *n* = 4 for SHAM groups, 5 for OVX groups. Black bar = 100 µm

GTT and ITT were performed to assess the effects of ActRIIB-Fc treatment on glucose clearance and insulin resistance. In GTT, there were no differences between the OVX and SHAM PBS groups suggesting that the effects of OVX on glucose tolerance are not acute (Fig. 6A). Interestingly, ActRIIB-Fc treatment in OVX mice resulted in significantly higher blood glucose values throughout the tolerance test (RM ANOVA *p* < 0.001) compared to the OVX PBS mice. A similar trend was also observed in the ActRIIB-Fc-treated SHAM mice at the later time points of the tolerance test compared to the SHAM PBS (RM ANOVA *p* = 0.086). The area under the curve was also quantified and showed treatment with ActRIIB-Fc in OVX mice to significantly impair glucose clearance while an analogous tendency was seen in ActRIIB-Fc-treated SHAM mice (Fig. 6B). These results suggest that treatment with ActRIIB-Fc impairs glucose clearance especially in OVX mice but may alter glucose tolerance also in intact mice.

Differences in ITT results were only noted at the late time points of the test as insulin resistance was only mildly increased in OVX mice regardless of ActRIIB-Fc or PBS treatment. However, these changes were not statistically significant (Fig. 6C). Furthermore, the AUC of the ITT results showed no significant differences between the groups (Fig. 6D). We did not observe any difference in fasting insulin levels between any of the groups indicating that basal insulin production was not altered by either OVX or treatment with ActRIIB-Fc (data not shown). Taken together, these data suggest that ActRIIB-Fc treatment induces a modest glucose intolerance, perhaps due to slight insulin resistance.

### ActRIIB-Fc Induces Hepatic Steatosis

To investigate the putative mechanisms underlying the impaired glucose clearance, we analyzed liver histology. (Fig. 6E–H). There were no major differences in the overall morphology of hepatocytes as seen in the H&E staining (data not shown). However, Oil red O-staining demonstrated ActRIIB-Fc to greatly induce hepatic accumulation of lipid droplets compared to the PBS-treated controls in both SHAM and OVX mice (ANOVA Treatment *p* < 0.001) (Fig. 6I). These data suggest that ActRIIB-Fc induces liver steatosis. Thus, although ActRIIB-Fc treatment does induce an increase in lean mass and reduces WAT adipocyte size, it leads to lipid accumulation in the liver.

## Discussion

In this study, we evaluated the effects of inhibition of ActRIIB ligands on bone mass and body composition in OVX mouse model using a soluble activin type IIB receptor fusion protein ActRIIB-Fc. Our initial hypotheses were confirmed as ActRIIB-Fc treatment was able to rescue the bone loss induced by ovariectomy as well as to increase bone mass in the SHAM group compared to their PBS-treated controls. We also confirmed that ActRIIB-Fc treatment resulted in lower systemic adiposity and increased lean mass in both SHAM and OVX mice when compared to their PBS-treated controls. Based on these results, we expected ActRIIB-Fc treatment to enhance metabolic parameters including glucose tolerance. Interestingly, despite increased lean mass and decreased fat mass, ActRIIB-Fc treatment resulted in impaired glucose clearance in OVX mice and a similar trend was noticed in SHAM mice. Insulin tolerance test showed OVX to cause mild insulin resistance and treatment with ActRIIB-Fc did not affect this in either SHAM or OVX mice. Finally, analyses of liver histology showed increased adipocyte infiltration and liver steatosis due to ActRIIB-Fc treatment.

The relationship between activins and fat tissue is not well documented. Zaragosi et al. [31] suggested that adipogenesis is highly dependent on a self-renewing pool of adipose progenitors and that activin A regulates these cell by promoting their proliferation but inhibiting their differentiation. In addition, treatment with a specific activin A-antibody suppressed the proliferation of these cells. Therefore, activin A could be a critical regulator of the adipose precursor pool [32]. The relationship between myostatin, another ligand bound by ActRIIB-Fc, and adiposity is also complex as *MSTN*^*−/−*^ mice have less adipose tissue possibly due to an indirect mechanism induced by increased muscle mass [33]. Furthermore, there have been some discrepancies between studies concerning the effects of ActRIIB-Fc on WAT accumulation and obesity. Akpan et al. [24] found that treatment with RAP-031, the murine homolog of the soluble ActRIIB-Fc fusion protein, reduced total body fat in healthy mice fed with either normal chow or high fat diet. Similarly, Koncarevic et al. demonstrated that ActRIIB-Fc treatment decreases body fat accumulation and reduced adipocyte size in an orchiectomy (ORX) mouse model [25]. Conversely, McPherron et al. reported that ActRIIB-Fc treatment did not lead to decreased fat mass in obese mice [34]. In this study, however, the mice had a threefold higher starting fat weight compared to the mice in other studies and the results could favor the hypothesis of activins/myostatin regulating the precursor pool of adipocytes instead of affecting already accumulated fat depots.

Our results are in concordance with previous reports of ActRIIB-Fc acting as an anti-adiposity agent as ActRIIB-Fc treatment reduced systemic fat accumulation in the OVX group compared to the PBS-treated mice. Similar but more modest changes were also noted in the SHAM mice. In addition, we showed ActRIIB-Fc to site-specifically reduce WAT accumulation in the gonadal and mesenteric fat pads compared to PBS-treated mice in the OVX groups as well to attenuate OVX-induced adipocyte hypertrophy. Therefore, our results support the hypothesis that inhibition of ActRIIB ligand signaling decreases fat accumulation at specific fat depots to an extent that it results in decreased overall adiposity.

The inhibition of activin type II receptor ligands has emerged as a potent therapeutic agent for treating musculoskeletal disorders by improving both muscle and bone mass. Our µCT analyses showed a robust response to ActRIIB-Fc as it not only restored bone mass to its original level in the long bones and axial skeleton but greatly surpassed it by improving bone volume in a manner similar to what has been reported for ActRIIA-Fc in OVX mice [22]. The increased bone mass was also confirmed in our histomorphometric analyses. Mechanistically, this appeared to be at least in part due to the anti-resorptive effect of ActRIIB-Fc indicated by the decreased osteoclast surface and number in histomorphometry as well as decreased expression of osteoclast-related genes in our gene expression analyses. Interestingly, we also observed increased expression of osteoblastic gene Runx2 and some indications for increased osteoblast function in histomorphometry in ActRIIB-Fc-treated OVX mice suggesting that treatment with ActRIIB-Fc could have a dual effect in bone, acting as both as an anabolic and anti-resorptive agent. Despite the potent changes in bone volume in both OVX and SHAM mice, we did not find any significant differences in serum markers of bone formation (P1NP) and resorption (CTX) due to ActRIIB-Fc treatment at the end of the study (data not shown). The same observation has been made previously as well [16]. One explanation could be that the changes in P1NP and CTX-levels might peak during the first few weeks of the treatment when the effects of ActRIIB-Fc on bone metabolism appear to be most prominent [14]. Similarly, this conjecture could also help explain the lack of significant changes in osteoblast-related parameters in our histomorphometrical analysis. Therefore, a future study where samples of ActRIIB-Fc-treated animals would be collected from early time points could help elucidate this issue.

The effects of estrogen deficiency on glucose metabolism are not entirely clear. Current concepts suggest estrogen to stimulate glucose uptake in skeletal muscle through ER-α signaling. In fact, menopause-induced glucose intolerance is now thought to be the result of a low-grade inflammatory state, in part due to the increased accumulation of radical oxygen species (ROS) caused by estrogen deprivation [35, 36]. Interestingly, despite increased lean/muscle mass, which is a major factor in insulin-mediated glucose uptake and thus thought to enhance glucose clearance [37], our results showed ActRIIB-Fc to significantly induce glucose intolerance especially in OVX mice with a trend also in SHAM mice. This is in agreement with the recent report demonstrating that treatment with ActRIIB-Fc worsened the hyperglycemia in mice with streptozotocin-induced diabetes [38]. Activins, especially activin B, play important roles in the genesis and function of pancreatic islets—and beta cells and therefore could be potent regulators of glucose metabolism. Thus, our findings could be partly explained by the impaired pancreatic islet cell function and subsequently reduced insulin production due to inhibition of activin A and B, resulting in slower glucose clearance. This hypothesis is supported by a previous study, in which transgenic expression of ActRIIB induced pancreatic islet hypoplasia and reduced glucose tolerance [39]. However, our results showed no differences in fasting insulin levels (data not shown) or insulin resistance suggesting that glucose tolerance is impaired by another mechanism as suggested previously [38]. Conversely, Akpan et al. demonstrated ActRIIB-Fc to increase insulin’s effect to suppress glucose production possibly due to enhanced adiponectin secretion [24].

Another culprit for the impaired glucose clearance could be perturbations in liver homeostasis. The specific role of activins in hepatocytes is incompletely understood as activin A has been suggested to both protect hepatic tissue from adipose accumulation [40] as well as induce hepatic steatosis in activin-antagonizing follistatin-deficient mice [41]. In contrast to the report from Koncaveric et al., where they reported ActRIIB-Fc to decrease liver adiposity in ORX mice, we found ActRIIB-Fc to robustly increase lipid droplet accumulation in the liver. The development of hepatosteatosis could at least in part explain the impaired glucose tolerance in ActRIIB-Fc-treated mice due to reduced hepatic insulin sensitivity. Our results also suggest an interesting lipodystrophic effect as it is possible that there may also be some transitioning of lipids from other fat depots to the liver. It is also plausible to speculate whether the impaired glucose clearance precedes hepatic steatosis as shown before [42].

There is an urgent need for new treatment options for postmenopausal osteoporosis and the metabolic sequelae associated with it. Loss of bone mass and strength are correlated with higher fracture risks, which lead to periods of immobilization and increased morbidity and mortality. Obesity is strongly linked to metabolic disorders and can manifest also in cardiovascular disorders. Here, we demonstrate that ActRIIB-Fc treatment attenuates bone loss and fat accumulation in an OVX mouse model. Surprisingly, ActRIIB-Fc increased liver adiposity, which could contribute to the observed impaired glucose clearance. Collectively, we confirm ActRIIB ligands to be important regulators of muscle, bone, and fat tissues but also to seem to play a role in regulating liver adiposity. Further studies are needed to identify the specific ligands by which ActRIIB-Fc regulates target tissue phenotype before progressing with further clinical trials with these agents for the treatment of musculoskeletal disorders.
